# Single radiation exposure induces gut microbiota dysbiosis and decreases short-chain fatty acid metabolism and intestinal barrier integrity in mice

**DOI:** 10.3389/fcimb.2025.1654976

**Published:** 2025-09-17

**Authors:** Mineon Park, You Yeon Choi, Yanghee Lee, Minsu Cho

**Affiliations:** 1 Laboratory of Biological Dosimetry, National Radiation Emergency Medical Center, Korea Institute of Radiological & Medical Sciences (KIRAMS), Seoul, Republic of Korea; 2 Department of Convergence Korean Medical Science, College of Korean Medicine, Kyung Hee University, Seoul, Republic of Korea

**Keywords:** single and fractionated radiation responses, gut microbiota, SCFAs, intestinal barrier dysfunction, dysbiosis

## Abstract

Ionizing radiation causes biological damage, including DNA damage, inflammation, and tissue homeostasis disruption. The gastrointestinal tract, which harbors diverse gut microbiota, is particularly susceptible to radiation-induced injury and intestinal barrier dysfunction. We aimed to investigate the effects of single and fractionated radiation exposures on gut microbiota diversity and short-chain fatty acid (SCFA) metabolism. Mice were exposed to a single dose (1 Gy, one exposure; dose rate: 2.6 Gy/min) or fractionated doses (1 Gy accumulated over 75 fractions, 6.7 mGy/min for 2 min per session). *In vitro*, differentiated Caco-2 monolayers were used to assess radiation-induced tight junction disruption and reactive oxygen species (ROS) production. Single radiation exposure induced a stronger response than fractionated exposure, as evidenced by increased DNA damage foci, altered blood profiles, and elevated inflammatory cytokines. Gut dysbiosis was more pronounced in the single-radiation group, characterized by an increased Firmicutes/Bacteroidetes ratio and reduced microbial diversity. SCFA analysis revealed considerable reductions in acetic and propionic acid levels in the single-radiation group compared to those in the control and fractionated groups. The expression of the SCFA-sensing receptors GPR41 and GPR43 was markedly downregulated in the single-radiation group. Tight junction proteins (*TJP1, CLDN1, CLDN3*, and *OCLN*) were markedly decreased, indicating compromised intestinal barrier integrity and increased permeability both *in vivo* and *in vitro*. Single radiation exposure caused greater gut microbiota and metabolic disruptions than fractionated radiation exposure, emphasizing the distinct effects of each type and the critical roles of gut microbiota and SCFAs in radiation-induced intestinal damage.

## Introduction

Radiation exposure is a significant environmental factor that can disrupt the composition and functionality of the gut microbiota in animals, leading to alterations in intestinal homeostasis and microbial gene expression. These changes may influence host metabolism, immune responses, and overall gut function (Fernandes et al., 2021). Given the growing concerns about radiation contamination from nuclear activities, medical applications, and occupational exposure, understanding how radiation affects gut microbiomes is essential for elucidating host-microbe interactions under environmental stress.

Ionizing radiation leads to acute and chronic toxicity in the body, including DNA damage, inflammation, tissue damage, and increased cancer risk (Yahyapour et al., 2018). Acute radiation syndrome occurs when the entire body or a significant portion is exposed to radiation doses exceeding 1 Gy (López and Martín, 2011). High-dose ionizing radiation is associated with acute radiation syndromes, including hematopoietic, gastrointestinal, and neurovascular syndromes, following partial or total body radiation exposure (Christensen et al., 2014; Macià I Garau et al., 2011; Pariset et al., 2021). The gastrointestinal tract, the largest reservoir of the gut microbiota, is particularly vulnerable to radiation damage (Guo et al., 2020). Clinical observations of patients receiving radiation therapy further emphasize this susceptibility. Patients receiving abdominal or pelvic radiation therapy frequently report GI symptoms, such as diarrhea, malabsorption, and nausea, which indicate structural and functional disruption of the intestinal barrier (Andreyev, 2007). These findings highlight the role of the microbiome in mediating radiation-induced GI damage.

The biological effects of radiation exposure can vary significantly depending on whether the dose is a single dose or a fraction. Acute, single radiation exposure can lead to cellular damage, resulting in severe tissue injury. In contrast, fractionated radiation exposure provides intermittent recovery periods, allowing partial repair of damaged cells and tissues, which can modulate the severity of radiation-induced effects (McKelvey et al., 2018). Oxidative stress from single radiation exposure can weaken cellular defenses and induce rapid and dramatic changes in the gut microbiome compared to fractionated exposure (Lu et al., 2024). This suggests that the gut microbiome plays an important role in modulating the severity of radiation-induced damage.

Several studies have highlighted the important interplay between gut microbiota and radiation-induced responses. The gut microbiota influence host metabolism, immune regulation, and intestinal barrier integrity. Radiation-induced dysbiosis, characterized by decreased microbial diversity and altered community composition, exacerbates inflammation and impairs mucosal healing owing to decreased beneficial taxa and disruptions in key microbial metabolites, such as short-chain fatty acids (SCFAs) (Crawford and Gordon, 2005). SCFAs, such as acetic acid, propionic acid, and butyrate, are essential for maintaining gut health and alleviating inflammation. Radiation-induced SCFA disturbances worsen intestinal permeability and systemic inflammatory responses, thereby exacerbating radiation toxicity (Louis et al., 2014).

The study was designed as a practical reference point to compare the physiological differences between fractionated and single irradiation using the same cumulative dose. Our primary aim was to evaluate whether identical total doses, when delivered acutely or fractionated over time, would lead to distinct tissue responses and microbial–immune alterations. The total radiation dose of 1 Gy was chosen based on both scientific precedent and translational relevance. Importantly, 1 Gy is widely recognized as a threshold dose for triage assessment of acute radiation syndrome (ARS) in clinical and radiological emergency settings, according to international radiation protection guidelines (e.g., IAEA, CDC). This threshold reflects the point at which measurable biological effects, including hematopoietic suppression and gastrointestinal dysfunction, begin to manifest in exposed individuals (López and Martín, 2011; National Council on Radiation Protection and Measurements, 2001). Thus, the selected dose allows us to explore biologically meaningful alterations while avoiding severe lethality. In this context, we specifically compared how single and fractionated radiation exposures affect gut microbiota, intestinal barrier integrity, and short-chain fatty acid (SCFA) production, aiming to clarify their distinct physiological impacts.

## Materials and methods

### Animal model and irradiation

**Figure 1:**
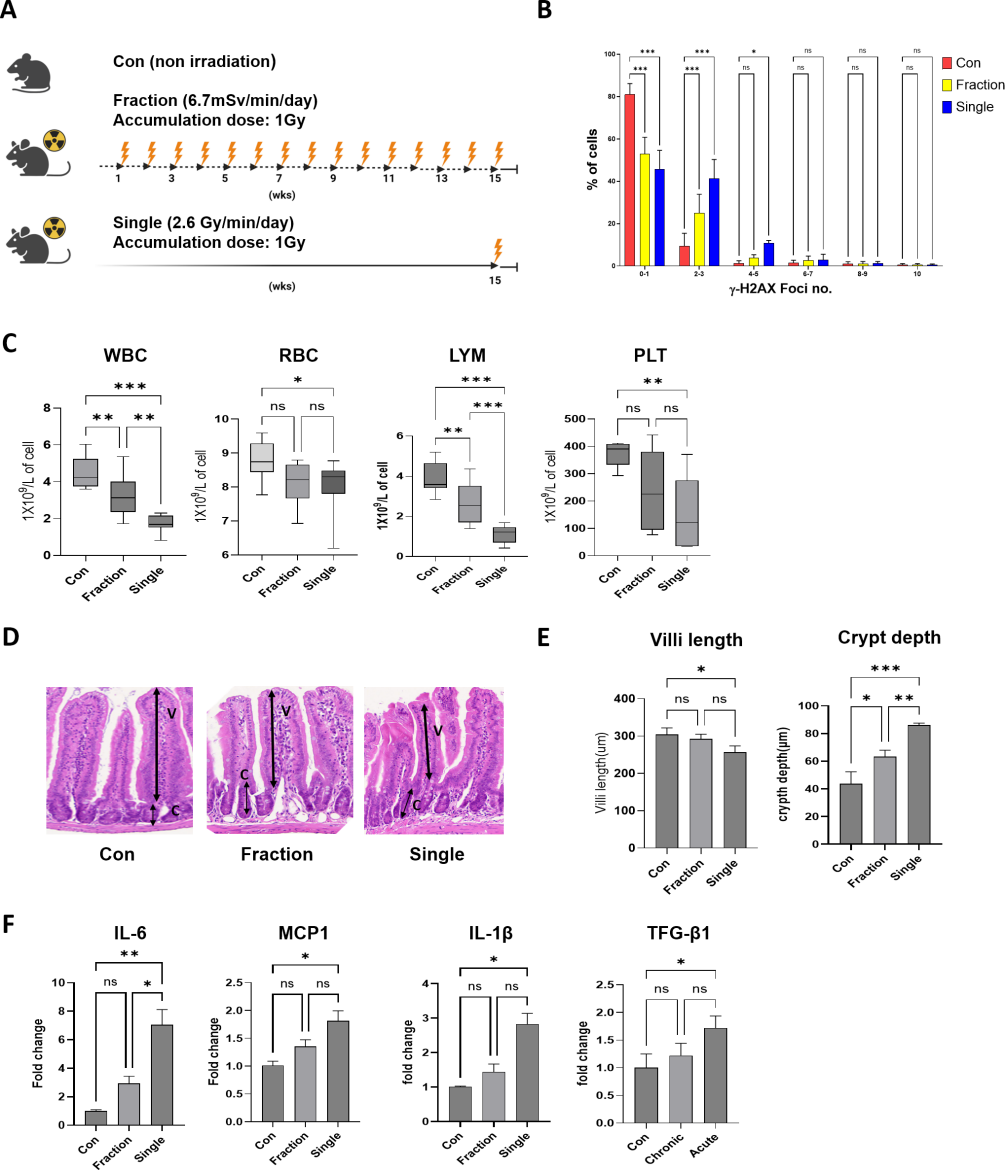
Analysis of radiation response to fractionated and single radiation exposures in mouse models. **(A)** Schematic representation of the experimental design for fractionated and single radiation exposures. Mice were exposed to whole-body radiation at a total dose of 1 Gy, either in fractions over 15 weeks or as a single exposure. The fractionated radiation group received a cumulative dose of 1 Gy over 75 sessions (dose rate: 6.7 mGy/min), whereas the single radiation exposure group received a single-dose exposure of 1 Gy (dose rate: 2.6 Gy/min). **(B)** Representative distribution of γ-H2AX foci numbers in mouse peripheral blood mononuclear cells. **(C)** Complete blood count analysis after radiation exposure in the Con, Fraction, and Single groups. **(D, E)** Histological assessment of intestinal tissues was performed using H&E-stained sections. Villi length and crypt depth were measured, and representative images of intestinal sections are provided. **(F)** Quantification of inflammatory cytokine mRNA expression levels, including IL-6, MCP-1, IL-1β, and TGF-β1, in mouse intestinal tissue using qRT-PCR. Data are presented as the mean ± standard deviation. Significant differences are indicated as ***p < 0.001, **p < 0.01, *p < 0.05 between groups. Con, non-irradiated control group; Fraction, fractionated radiation exposure group; Single, single radiation exposure group. ns = not significant.

Eight-week-old male C57BL/6 mice were purchased from Doo Yeol Biotech, Inc. (Seoul, Korea) and maintained under specific pathogen-free conditions. After a 1-week acclimation period, the mice were randomly divided into non-irradiated (control, Con), fractionated radiation exposure (Fraction), and single radiation exposure (Single) groups (n = 12 per group). Single irradiation was performed using a gamma-ray machine (BIOBEAM8000, 137Cs; dose rate: 2.6 Gy/min), and fractionated irradiation was performed using a different gamma-ray machine (MDI-KIRAMS 137, 137Cs; dose rate: 6.7 mGy/min) to irradiate the entire body of the mice. The single exposure group received a single 1 Gy dose on the final day of fractionated radiation. The fractionated exposure group received radiation 5 days per week for 2 min per day for 15 weeks, accumulating a total dose of 1 Gy (75 fractions, 6.7 mGy/min, 2 min per session). A schematic of the experimental design is shown in **Figure 1A**. The total dose of 1 Gy was selected based on previous studies investigating low-dose radiation biology (Morgan and Bair, 2013; Azzam et al., 2012). Fractionated exposure over 15 weeks was designed to mimic environmental or occupational low-dose radiation scenarios (Brooks and Couch, 2006; Tanaka et al., 2003; Gapeyev et al., 2015). The single-dose group was included to compare acute versus chronic responses, since prior reports demonstrate distinct biological outcomes depending on radiation delivery mode (Mitchel, 2006). Blood samples were collected from each mouse by cardiac puncture one week after radiation exposure and were immediately transferred into EDTA-coated tubes. Fecal samples were collected on day 7 post-exposure by placing each mouse in a sterile cage. All intestinal tissues were harvested on the day of sacrifice; those for histological analysis were fixed in paraformaldehyde, while those for molecular analyses were snap-frozen and stored at –80°C until use.

### Complete blood count analysis

All mice were placed under general anesthesia, and blood was collected via cardiac puncture into tubes prefilled with EDTA (K2EDTA, BD Microtainer #365974). A CBC was performed using a hematology analyzer (Abaxis/HM5), measuring white blood cells (WBC), red blood cells (RBC), lymphocytes (LYM), and platelets (PLT).

### γ-H2AX foci analysis

Radiation-induced DNA damage was assessed by quantifying γ-H2AX foci in mouse peripheral blood mononuclear cells. RBCs were lysed using eBioscience™ RBC lysis buffer (Invitrogen, Waltham, MA, USA) and fixed with 1× Lyse/fix solution (BD Phosflow™; BD Biosciences, San Jose, CA, USA). Fixed cells were permeabilized with 0.1% Triton X-100 (Sigma-Aldrich, St. Louis, MO, USA) for 10 min at room temperature (RT) and then stained with Alexa Fluor^®^ 488 Mouse anti-H2AX (pS139) antibody (clone N1–431, BD Pharmingen™; Becton Dickinson, Franklin Lakes, NJ, USA) for 1 h at RT. After washing with 1× phosphate-buffered saline (PBS), cells were stained with 20 μM DRAQ5™ (Thermo Fisher Scientific, Waltham, MA, USA) for 5 min at RT. γ-H2AX foci were quantified using imaging flow cytometry.

### Quantitative reverse transcription polymer chain reaction

We performed qRT‐PCR to measure mRNA expression levels. Total RNA was extracted from mice intestine tissues and Caco-2 cells using TRIzol reagent (Invitrogen) according to the manufacturer’s instructions. cDNA was synthesized using oligo-dT primers and AccuPower RT premix (Bioneer, Daejeon, Korea). The synthesized cDNA was amplified using a LightCycler 480 system (Roche, Basel, Switzerland) with specific primers. The cycling conditions were 95°C for 10 min, followed by 40 cycles at 95°C for 15 s and 60°C for 1 min. Data were analyzed using StepOne software version 2.2.2 (Applied Biosystems, Waltham, MA, USA). The mRNA expression levels were normalized to GAPDH, and fold changes were calculated using the 2^-ΔΔCt^ method. The primers used for qRT-PCR are listed in **Table 1**.

**Table 1:** List of qRT-PCR primer sequences used in this study.

Gene		Forward primer	Reverse primer
Mouse	*GAPDH*	5’-ATG CCA GTG AGC TTC CCG TTC AG-3’	5’-CAT CAC TGC CAC CCA GAA GAC TG-3’
	*IL-6*	5’-TAC CAC TTC ACA AGT CGG AGG C-3’	5’-CTG CAA GTG CAT CAT CGT TGT TC-3’
	*MCP1*	5’-GCA GCA GGT GTC CCA AAG AA-3’	5’-ATT TAC GGG TCA ACT TCA CAT TCA A-3’
	*IL-1β*	5’-TGG ACC TTC CAG GAT GAG GAC A-3’	5’-GTT CAT CTC GGA GCC TGT AGT G-3’
	*TGF-β1*	5’-ACT GGA GTT GTA CGG CAG TG-3’	5’-GGG GCT GAT CCC GTT GAT TT-3’
	*GPR41*	5’-GGG GTC GAT ACA AGA GT-3’	5’-CTG GCG GAG CTA CGT GCT-3’
	*GPR43*	5’-TTC TTA CTG GGC TCC CTG CC-3’	5’-TAC CAG CGG AAG TTG GAT GC-3’
	*GPR109*	5’-TCA GAT CTG ACT CGT CCA CC-3’	5’-CCA TTG CCC AGG AGT CCG AAC-3’
	*TJP1*	5’-GTT GGT ACG GTG CCC TGA AAG A-3’	5’-GCT GAC AGG TAG GAC AGA CGA T-3’
	*CLDN1*	5’-GGA CTG TGG ATG TCC TGC GTT T-3’	5’-GCC AAT TAC CAT CAA GGC TCG G-3’
	*CLDN3*	5’-TCA TCG TGG TGT CCA TCC TGC T-3’	5’-AGA GCC GCC AAC AGG AAA AGC A-3’
	*OCLN*	5’-TGG CAA GCG ATC ATA CCC AGA G-3’	5’-CTG CCT GAA GTC ATC CAC ACT C-3’
Human	*GAPDH*	5’-GGA CTC ATG ACC ACA GTC CAT GCC-3’	5’-TCA GGG ATG ACC TTG CCC ACA G-3’
	*TJP1*	5’-GTC CAG AAT CTC GGA AAA GTG CC-3’	5’-CTT TCA GCG CAC CAT ACC AAC C-3’
	*OCLN*	5’-ATG GCA AAG TGA ATG ACA AGC GG-3’	5’-CTG TAA CGA GGC TGC CTG AAG T-3’
	*CLDN1*	5’-GTC TTT GAC TCC TTG CTG AAT CTG-3’	5’-CTG TAA CGA GGC TGC CTG AAG T-3’

### DNA extraction and gut microbiome 16S ribosomal RNA gene analysis

Fecal DNA was extracted using the DNeasy PowerSoil^®^ Kit (Qiagen, Hilden, Germany) according to the manufacturer’s protocol. Extracted DNA was quantified using Quant-IT PicoGreen (Invitrogen). For microbiome analysis, the V3–V4 regions of the 16S rRNA gene were amplified according to the Illumina 16S Metagenomic Sequencing Library protocol. A total of 5 ng of genomic DNA (gDNA) was PCR-amplified using a 5× reaction buffer, 1 mM dNTP mix, 500 nM of universal forward and reverse primers, and Herculase II fusion DNA polymerase (Agilent Technologies, Santa Clara, CA, USA). The first PCR cycle conditions were as follows: initial denaturation at 95°C for 3 min, followed by 25 cycles at 95°C for 30s, 55°C for 30s, 72°C for 30s, with a final extension at 72°C for 5 min. PCR products were purified using AMPure beads (Agencourt Bioscience Corporation, Beverly, MA, USA). For final library construction, 2 µL of the first PCR product was subjected to a second PCR using Nextera XT Indexed Primer under the same conditions as the first PCR, except for 10 cycles. The PCR products were purified using AMPure beads. To ensure optimal cluster densities on Illumina sequencing platforms, accurate quantification of DNA library templates was performed using qPCR, following the Illumina qPCR Quantification Protocol Guide (KAPA Library Quantification Kit for Illumina Sequencing Platforms). Library quality was assessed using the TapeStation D1000 ScreenTape (Agilent Technologies). Paired-end sequencing (2 × 300 bp) was performed using a MiSeq™ sequencer (Illumina, San Diego, CA, USA).

### Sequencing data analysis

Paired-end FASTQ files were converted into QIIME2 artifacts for further analysis. Demultiplexed data were processed using the DADA2 algorithm, which included error correction and removal of rare taxa, to generate representative sequences and a feature table. Microbial classification of each representative sequence was confirmed by blasting against the 16S rRNA gene database. The Q2-Feature classifier, a Naive Bayes classifier trained based on the SILVA reference database (V3–V4 region; https://www.arb-silva.de/), was used to classify the dataset. The resulting feature table was used to generate a phylogenetic tree for downstream alpha and beta analyses. The “core metrics analysis” command was used to calculate Shannon diversity, Pielou’s evenness, observed operational taxonomic units, and Simpson’s index. Analysis of composition of microbiomes was used to verify the differences in feature composition between groups, and the results were visualized.

### Quantitative SCFA detection

SCFAs were quantitatively detected using gas chromatography-mass spectrometry (GC-MS). Fecal samples were mixed with 1 N HCl and an internal standard (0.1 M isobutanol), followed by vortexing for 10 min at RT. Extraction was performed using diethyl ether (2:1 ratio), and the mixture was centrifuged at 12,000 rpm for 5 min at 4°C to collect the supernatant. After collecting the supernatant, 1 µL was injected into a gas chromatograph-mass spectrometer (Hewlett Packard Model 7890) equipped with a DB-FATWAX Ultra Inert column. SCFAs were identified and quantified based on the retention time and standard calibration curves.

### Histopathological examination

For histological examination, formalin-fixed, paraffin-embedded small intestine tissues were cut into 3 μM sections, mounted on slides, dewaxed in xylene, and rehydrated in 30–100% ethanol. The sections were stained with hematoxylin and eosin (H&E). Stained slides were scanned using a slide scanner (MoticEasyScan Pro 6).

### Cell culture and irradiation

Human Caco-2 cells, derived from human colorectal adenocarcinoma, were maintained in Dulbecco’s Modified Eagle’s Medium (DMEM; Corning, Corning, NY, USA) supplemented with 10% fetal bovine serum (FBS; Gibco; Thermo Fisher Scientific) and 1% antibiotics. For *in vitro* studies of the intestinal barrier, we differentiated Caco-2 cells into an epithelial monolayer resembling that of enterocytes. Cells were cultured in a humidified incubator at 37°C with 5% humidity. Caco-2 cells were exposed to either fractionated or single radiation doses. The fractionated group was irradiated with 0.25 Gy/day for 4 days, totaling 1 Gy, whereas the single exposure group was irradiated with a single dose of 1 Gy. Both radiation exposures were performed using a gamma-ray machine (BIOBEAM8000, 137Cs; dose rate: 2.6 Gy/min).

### Reactive oxygen species analysis

ROS levels were assessed using a H_2_DCFDA staining kit (#ab113851; Abcam, Cambridge, UK) according to the manufacturer’s instructions. Caco-2 cells were exposed to radiation and incubated for 24 h before ROS measurement. Cells were treated with H_2_DCFDA (20 μM) in serum-free media at 37°C for 30 min in the dark. After staining, cells were washed with PBS, harvested, and resuspended in fresh PBS. The fluorescence intensity of oxidized H_2_DCFDA (excitation: 488 nm, emission: 525 nm) was quantified using imaging flow cytometry. Unstained cells served as negative controls for ROS validation.

### Immunocytochemical staining

Caco-2 cells were seeded onto four-well cell culture slides for immunocytochemical analysis. Cells were fixed with 4% paraformaldehyde, blocked with 3% bovine serum albumin (BSA), and permeabilized with 1% BSA and 0.1% Triton X-100 for 30 min at RT. Primary antibody ZO-1 (#339100; Thermo Fisher Scientific) was applied at a 1:1000 dilution in blocking buffer and incubated overnight at 4°C. The following day, cells were washed thrice with PBS and incubated with Alexa Fluor 488-conjugated anti-mouse IgG (Thermo Fisher Scientific) for 1 h at RT in the dark. After three washes with PBS, the cells were counterstained with DAPI for nuclear visualization and mounted using a fluorescence mounting medium. Fluorescent images were acquired using a Carl Zeiss Axioscope 5 fluorescence microscope.

### Exogenous SCFA supplementation

Caco-2 cells were irradiated with a single dose of 1 Gy (2.6 Gy/min) and subsequently treated with exogenous short-chain fatty acids (SCFAs). Cells were incubated with 2 mM sodium acetate (#CAS No. 127-09-3; SAMCHUN, Seoul, Korea), 2 mM sodium propionate(#Cat. No. P1880; Sigma-Aldrich St. Louis, MO, USA), or a mixture of both (2 mM each) for 24 h. Following treatment, cells were harvested for downstream analyses, including ROS measurement (H_2_DCFDA assay), qRT-PCR for tight junction genes (*Tjp1, OCLN, CLDN1*), and immunofluorescence staining of ZO-1.

### Statistics

Data are presented as the mean ± standard deviation. Statistical significance was set at *p* < 0.05. Comparisons between groups were performed using the Kruskal–Wallis test and one-way analysis of variance. Dunnett’s test was used for multiple comparisons against the control (Con), while Tukey’s *post-hoc* test was used for pairwise comparisons among all groups (Con, Fraction, and Single). Correlation heatmap analyses were performed using Pearson’s correlation coefficient. Statistical analyses were performed using GraphPad Prism version 10.4.1 (GraphPad Inc., CA, USA), and SPSS Statistics version 30 (IBM Corp., NY, USA).

## Results

### Single-dose radiation causes greater DNA damage, hematopoietic suppression, and intestinal injury

To examine the differential biological effects of single and fractionated radiation exposures, the mice were assigned to three groups (Con, Fraction, and Single). γ-H2AX fluorescence intensity and foci number, markers of DNA double-strand breaks, were considerably increased in peripheral blood mononuclear cells from the Single group compared to those in the Fraction and Con groups (**Figure 1B**; **Supplementary Figure S1**). CBC analysis revealed that WBC, RBC, LYM, and PLT counts were markedly reduced in the Single group compared with those in the Con group. Additionally, WBC and PLT levels were notably lower in the Fraction and Single groups than in the Con group, with the Single group showing a more marked reduction compared to the Fraction group. There were no statistically significant differences in the RBC and PLT counts between the Con and Fraction groups or between the Fraction and Single groups (**Figure 1C**). Histological analysis of the small intestine showed that the villus length was markedly reduced in the Single group compared to that in the Con group, and there was no significant difference between the Con and Fraction groups or between the Fraction and Single groups. In both radiation exposure groups, the crypt depth considerably increased compared to that in the Con group; in particular, the increase was greater in the Single group than in the Fraction group (**Figures 1D, E**). Furthermore, the expression levels of inflammatory cytokine (interleukin [IL]-6, monocyte chemoattractant protein-1 [MCP-1], IL-1β, and transforming growth factor-beta 1 [TGF-β1]) mRNAs in mouse intestinal tissues did not exhibit significant differences between the Fraction and Con groups. However, all cytokine mRNAs were upregulated in the Single group compared to those in the Con group, with IL-6 exhibiting more than a six-fold increase (**Figure 1F**). These results suggest that single radiation exposure induces greater biological damage than fractionated exposure.

### Single-dose irradiation markedly disrupts gut microbiota composition

**Figure 2:**
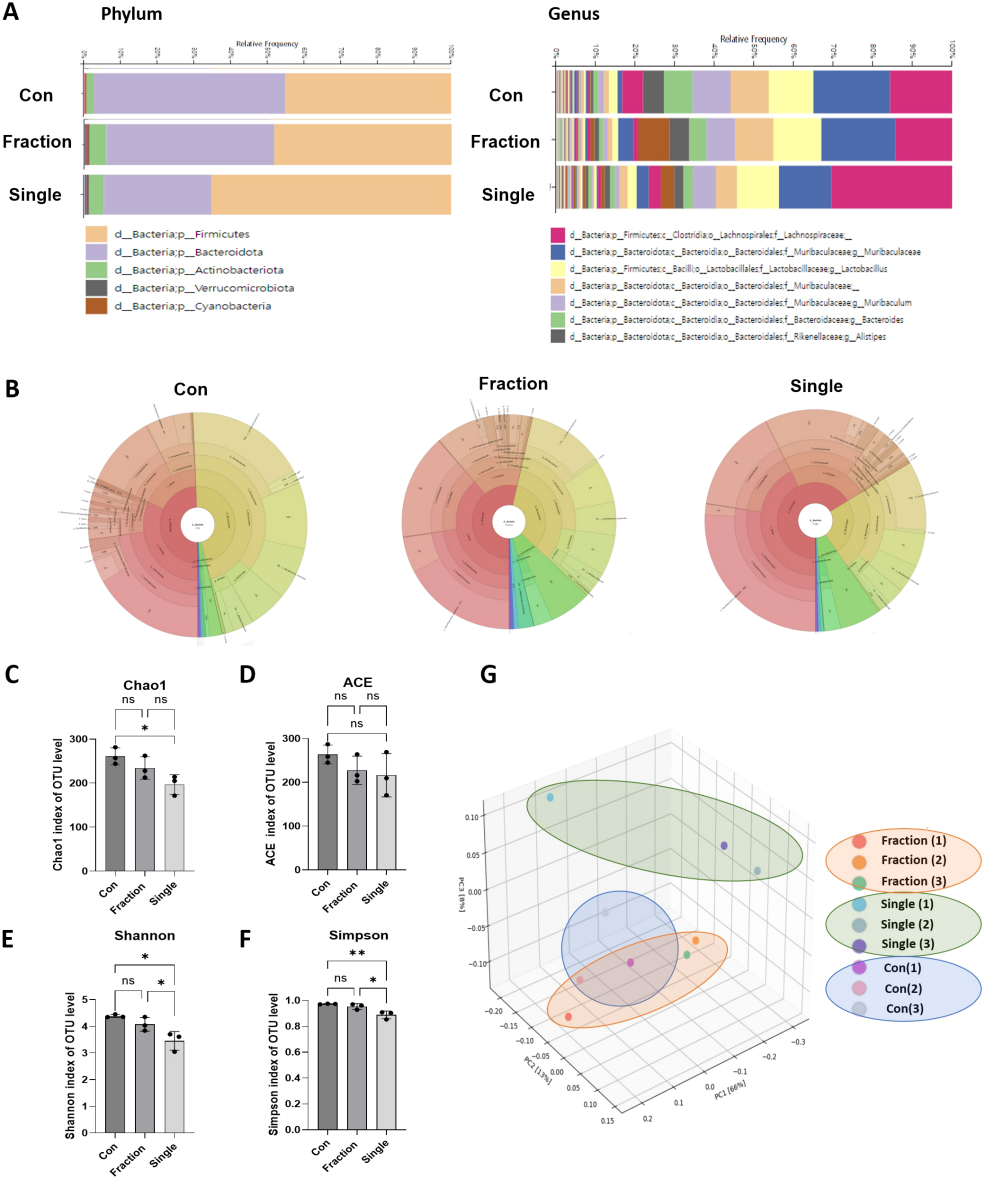
16S rRNA gene sequencing analysis of fecal DNA from fractionated and single radiation-exposed mice. **(A)** Comparison of the relative abundance bacterial taxa at the phylum and genus levels in the Con, Fraction, and Single groups. **(B)** Krona plots illustrating the microbial composition in the Con, Fraction, and Single groups. **(C-F)** Alpha diversity analysis of the gut microbiome, represented by Chao1, ACE, Shannon, and Simpson diversity indices for each group. **(G)** Principal coordinate analysis of fecal samples collected from irradiated mouse models. n = 12 mice per group, for 16S rRNA analysis, 3 mice were randomly selected per group. Data are presented as the mean ± standard deviation. Data was derived from three biological replicates, each with three technical replicates. Significant differences are indicated as *p < 0.05 between groups. Con, non-irradiated control group; Fraction, fractionated radiation exposure group; Single, single radiation exposure group. ** = p < 0.01, ns = not significant.

Gut microbiota composition was analyzed from fecal samples of mice in each irradiation group using bacterial 16S rRNA gene v3–v4 amplicon sequencing. At the phylum level, *Firmicutes*, *Bacteroidota*, and *Actinobacteriota* were the most abundant taxa (**Figure 2A**). The *Firmicutes/Bacteroidetes* (F/B) ratio differed remarkably among the groups, with the Single group showing a three-fold increase compared to the Con group and a two-fold increase compared to the Fraction group. No significant differences were observed between the Con and Fraction groups (**Supplementary Figure S2**). The relative abundance of *Firmicutes* was 49% in the Con group, 53% in the Fraction group, and 66% in the Single group, showing a marked increase in the Single group compared to the Con group. The relative abundance of *Bacteroidota* was 47% in the Con group, 34% in the Fraction group, and 23% in the Single group, showing a marked decrease in the Single group compared to the Con group. At the genus level, *Lachnospiraceae, Muribaculaceae, Lactobacillus, Muribaculum, Bacteroides, Alistipes, Turicibacter, Faecalibaculum*, and *Bifidobacterium* were the dominant genera. The relative abundance of *Muribaculaceae* decreased in the Single group compared to that in the Con group, reaching 15%, 14%, and 10% in the Con, Fraction, and Single groups, respectively. In contrast, *Lachnospiraceae* abundance increased in the Single group (32%) compared to that in the Con (15%) and Fraction (14%) groups. Krona charts showed more pronounced changes in community composition after single irradiation, whereas the Con and Fraction groups showed similar compositions. Notably, the imbalance in *Firumicutes* (pink) and *Bacteriodota* (yellow) was more prominent in the Single group, whereas the Con and Fraction groups showed similar compositions (**Figure 2B**). The Chao1 index markedly decreased in the Single group compared to that in the Con group, with no significant differences observed between the Con and Fraction groups or between the Fraction and Single groups. The abundance-based coverage estimator (ACE) index showed no significant differences among the groups. Additionally, the Shannon and Simpson diversity indices and richness were lower in the Single group compared to those in the Con and Fraction groups, with no significant differences between the Con and Fraction groups (**Figures 2C-F**). Furthermore, beta diversity, which indicates the difference in the composition of the intestinal microbial community between groups, showed that the distance between the Con and Fraction groups was similar. Conversely, the Single group exhibited a greater distance than the other groups according to the principal coordinate analysis, indicating a distinct microbial community structure (**Figure 2G**). These results suggest that single radiation exposure induces microbial dysbiosis, characterized by reduced and altered gut microbiota composition.

### Single-dose and fractionated radiation induce distinct changes in gut microbiota composition

**Figure 3:**
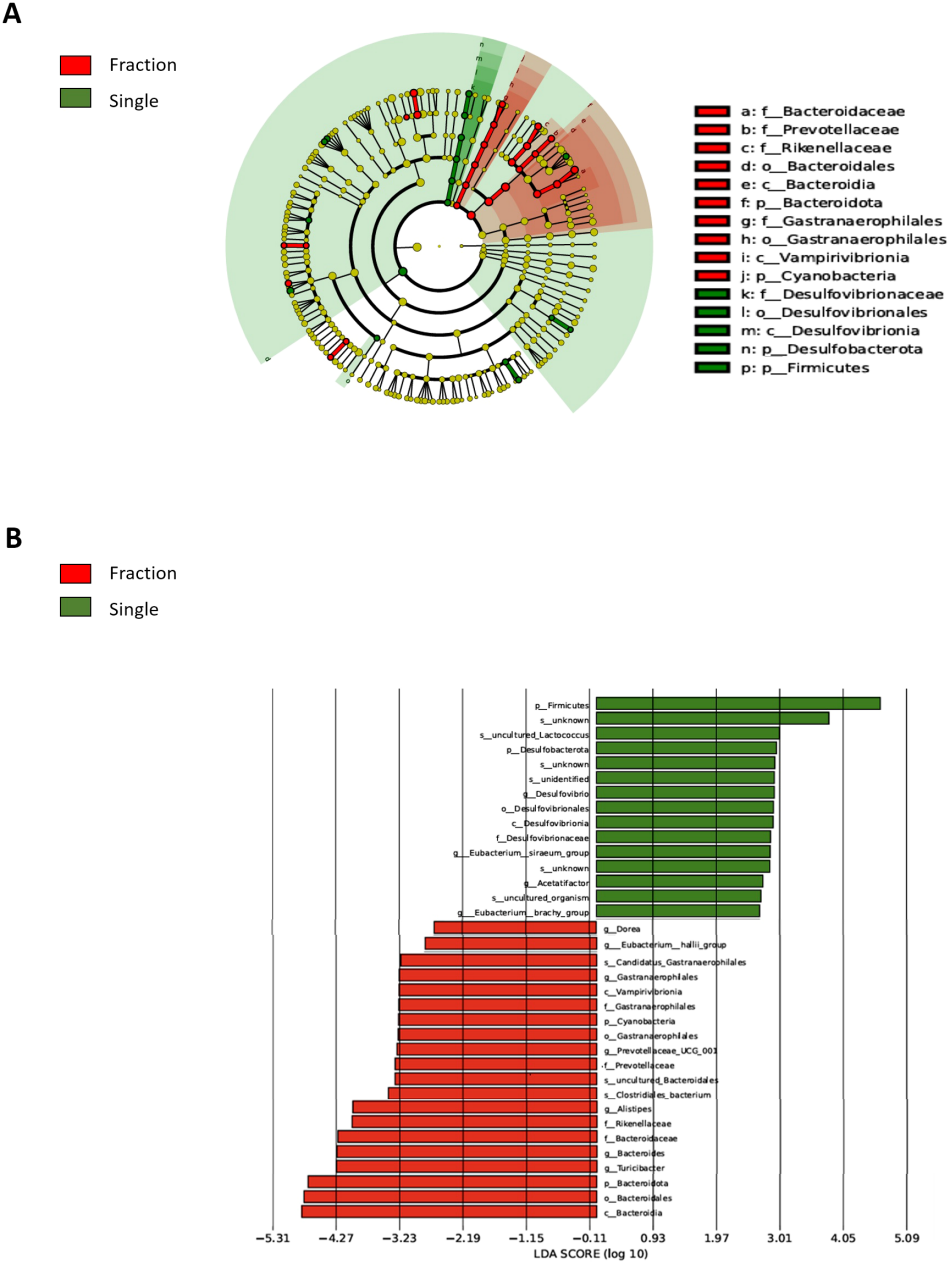
Comparison of differentially abundant microbial taxa in the fecal samples from single and fractionated radiation-exposed mice. **(A)** Evolutionary branch diagram (LEfSe analysis) showing differentially abundant microbial taxa between the fractionated and single radiation exposure groups. Taxa enriched in the Fraction group are highlighted in red, while those enriched in the Single group are highlighted in green. **(B)** LEfSe-based LDA plot comparing differentially abundant microbial taxa in fecal DNA from the Fraction and Single radiation exposure groups. Red bars represent taxa enriched in the Fraction group, while green bars indicate taxa enriched in the Single group. Con, non-irradiated control group; Fraction, fractionated radiation exposure group; single, single radiation exposure group.

Linear discriminant analysis Effect Size (LEfSe)-based Linear discriminant analysis (LDA) was performed to identify the key differences in the gut microbiome composition between the Fraction and Single groups. A taxonomic map of the gut microbiota structure (**Figure 3A**) highlights the most pronounced taxonomic differences between fractionated and single radiation exposures at the same dose. LEfSe-based LDA score analysis (**Figure 3B**) confirmed statistically significant differences in microbial abundance between the two groups (*p* < 0.05). In the Single group, we observed a higher relative abundance of *Firmicutes* (*p.*), *Lactococcus* (*spp*.), Desulfovibrionaceae (f.), Desulfovibrionales (*o*.), Desulfovibrionia (*c*.), and Desulfobacterota (p.). Notably, Desulfovibrionaceae and Desulfobacterota are sulfate-reducing bacteria (SRB) known to be associated with increased gut inflammation and epithelial barrier dysfunction. Conversely, the Fraction group exhibited a higher abundance of *Bacteroidota (p.), Bacteroidaceae (f.), Prevotellaceae (f.), Rikenellaceae (f.), Bacteroidia (c.), Bacteroidales (o.), Alistipes* (*g*.), *Turicibacter* (*g*.), *Gastranaerophilales (o.), Vampirivibrionia (c.)*, and *Cyanobacteria (p.)*. Many of these taxa, including *Prevotellaceae* and *Bacteroidota*, are known to produce SCFAs, which play protective roles in maintaining gut homeostasis and immune regulation. These findings suggest that single radiation exposure may contribute to an increase in inflammation-associated microbial taxa, whereas fractionated radiation exposure may favor the retention of SCFA-producing bacteria, potentially mitigating radiation-induced dysbiosis.

### SCFA levels and SCFA-sensing receptor expression are decreased after single-dose

**Figure 4:**
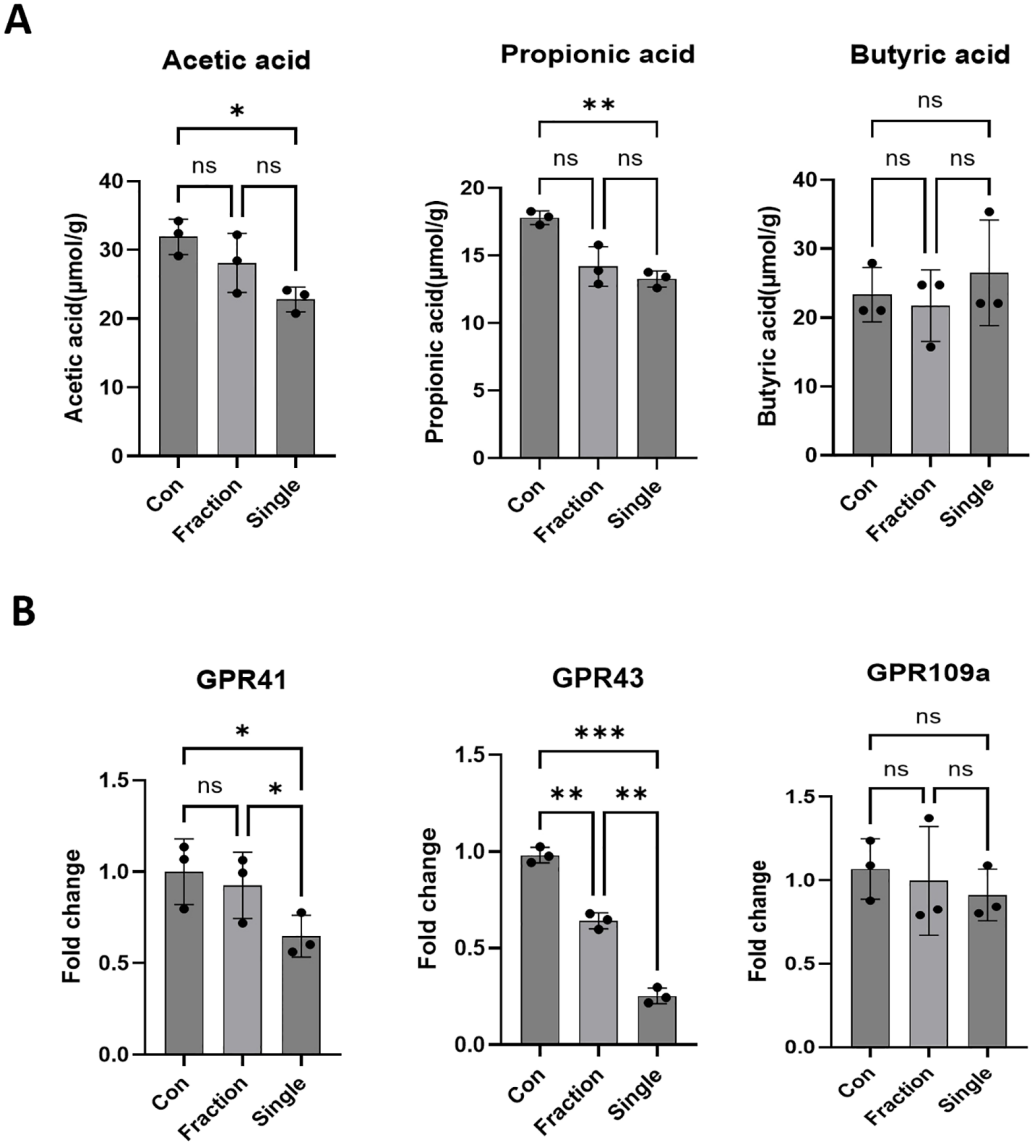
Alterations in SCFAs and SCFA-sensing receptor expression following fractionated and single radiation exposure. **(A)** Quantitative analysis of SCFAs, including acetic, butyric, and propionic acid, in fecal samples from the Con, Fraction, and Single groups using GC-MS. **(B)** Expression levels of SCFA-sensing receptors, specifically GPR41, GPR43, and GPR109, in intestinal tissues of mice from the Con, Fraction, and Single groups were analyzed by qRT-PCR. Data are presented as the mean ± standard deviation. Significant differences are indicated as ***p < 0.001, **p < 0.01, *p < 0.05 between groups. Con, non-irradiated control group; Fraction, fractionated radiation exposure group; Single, single radiation exposure group. ns = not significant.

We investigated how radiation-induced changes in the gut microbiota affect the expression of SCFAs and SCFA-sensing receptors. SCFAs, primarily produced through microbial fermentation of dietary fiber, play a crucial role in host metabolism, immune regulation, and intestinal homeostasis, particularly after radiation exposure (Eaton et al., 2022). SCFA analysis revealed a marked reduction in acetic acid and propionic acid levels in the Single group compared to those in the Con group, whereas no significant differences were observed between the Con and Fraction groups. Butyrate levels did not differ significantly between groups (**Figure 4A**). One of the key biological functions of SCFAs is the activation of G protein-coupled receptors (GPCRs), which are essential for host health and the regulation of cellular signaling (Priyadarshini et al., 2018). The Single group exhibited notably lower expression levels of GPR41 and GPR43 than the Con and Fraction groups, whereas GPR109a expression remained unchanged across the groups (**Figure 4B**). These findings suggest that single radiation exposure leads to a reduction in SCFAs, accompanied by decreased expression of SCFA-sensing GPCRs, potentially contributing to radiation-induced gut dysfunction.

### Single-dose irradiation disrupts SCFA–GPR correlations observed in SCFA-producing tax

**Figure 5:**
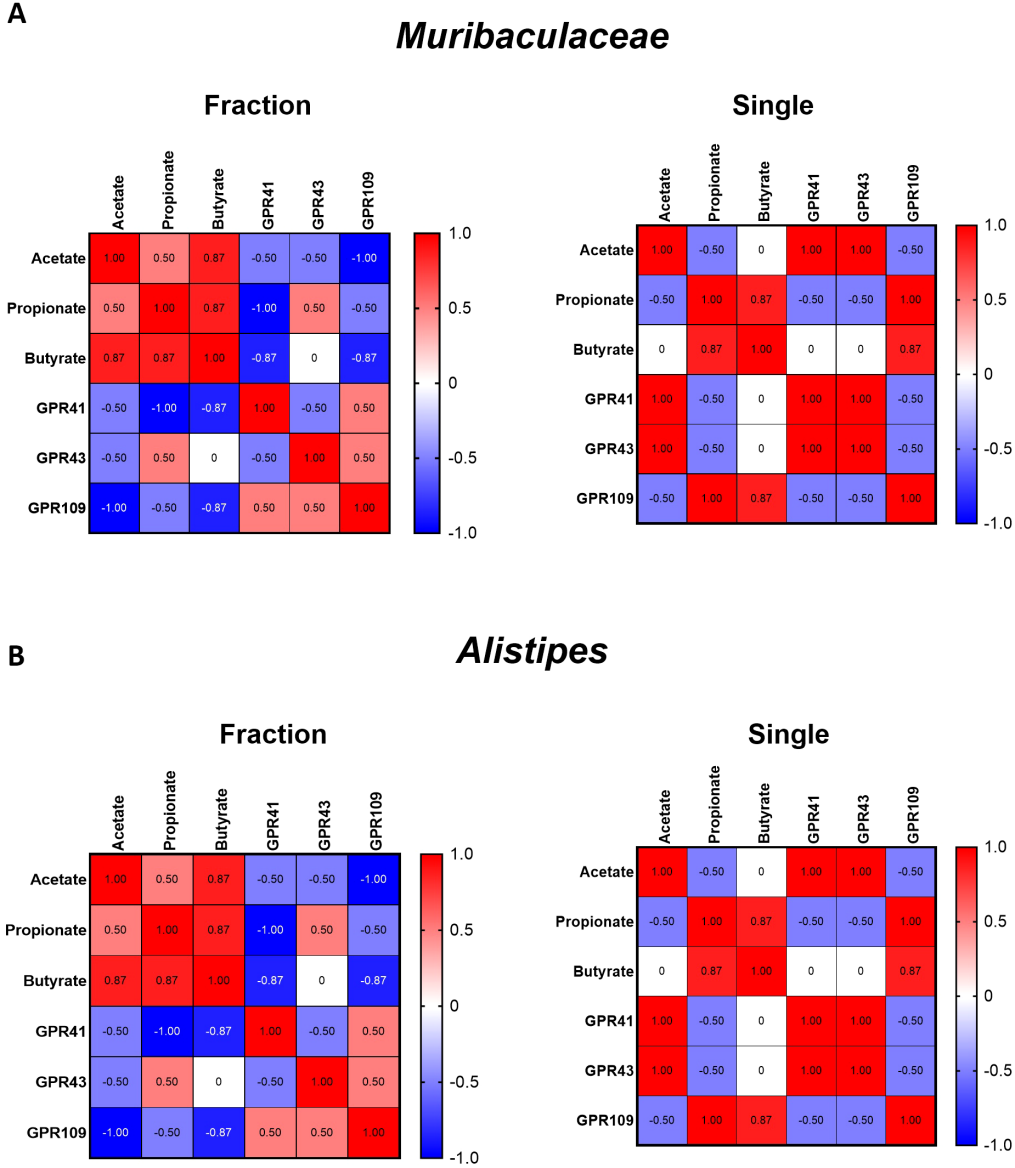
Correlation analyses between SCFA-producing bacteria, metabolites, and SCFA-sensing receptors under different irradiation regimens. **(A)** Heatmaps showing correlation patterns of Muribaculaceae abundance with major SCFAs (acetate, propionate, butyrate) and their receptors (GPR41, GPR43, GPR109) in the fractionated (left) and single-dose (right) groups. **(B)** Heatmaps showing correlation patterns of Alistipes abundance with SCFAs and receptors under fractionated (left) and single-dose (right) conditions. The heatmap provides a visual representation of the Pearson correlation coefficients between each pair of parameters. Color scale represents correlation coefficients (red = positive, blue = negative, white = no correlation; range –1.0 to +1.0).

Correlation heatmaps revealed distinct patterns of the microbiota–metabolite–SCFA receptor signaling axis between the radiation regimens (**Figure 5**). In the fractionated group, *Muribaculaceae* abundance correlated strongly with acetate (r = 0.87, p < 0.01), propionate (r = 0.86, p < 0.01), and GPR41 (r = 0.88, p < 0.01) and GPR43 (r = 0.85, p < 0.01) expression. Similarly, *Alistipes* showed significant positive correlations with acetate (r = 0.84, p < 0.05), propionate (r = 0.83, p < 0.05), and GPR41/43 (r = 0.85 and r = 0.82, respectively). In contrast, in the single-dose group, these correlations were markedly weakened (r values close to 0) or reversed (negative r values), indicating disruption of SCFA-mediated GPR signaling pathways.

### Single radiation exposure induces dysfunction of the intestinal tight junction barrier

**Figure 6:**
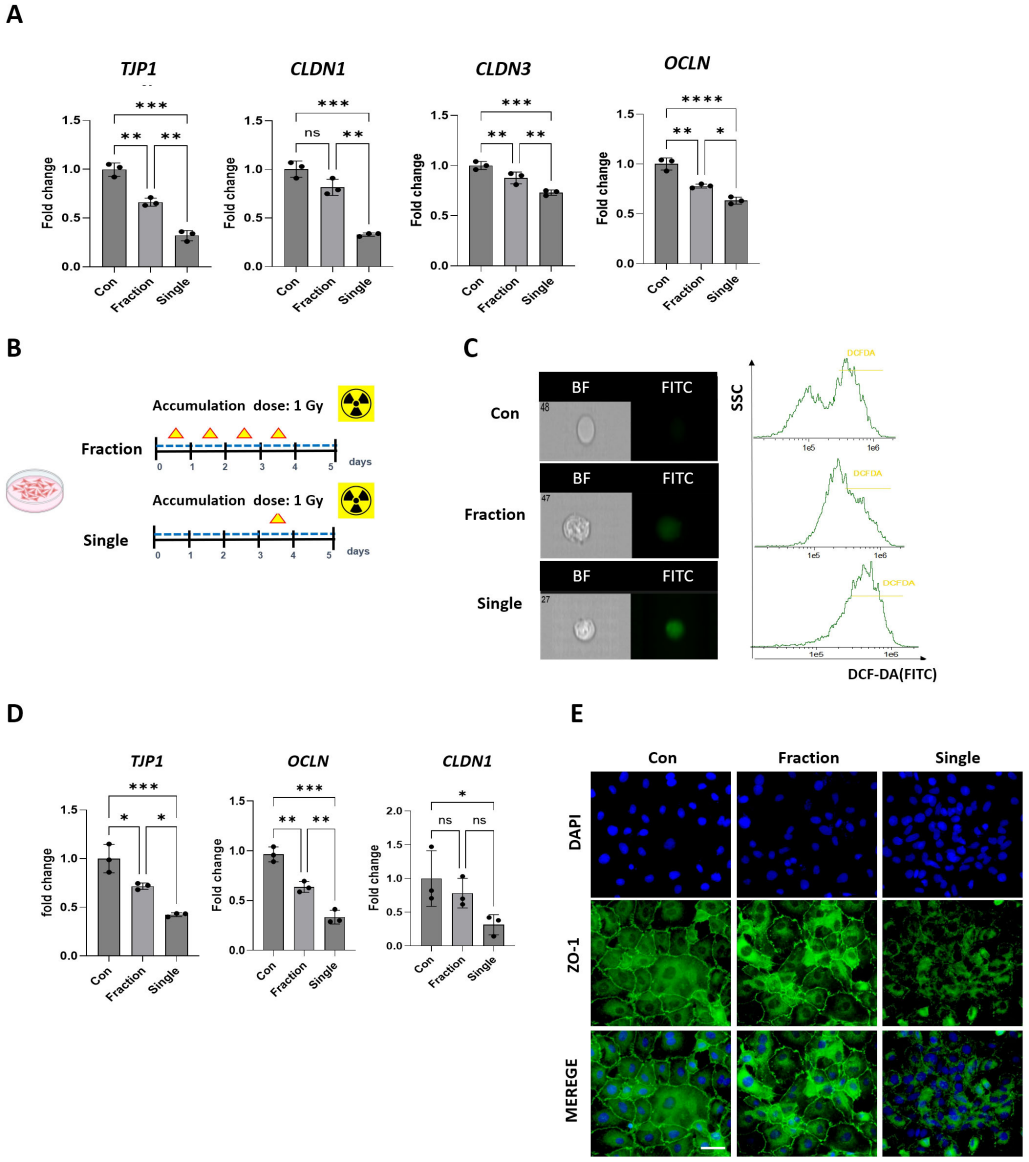
Analysis of tight junction-related factors following fractionated and single radiation exposure. **(A)** mRNA level of tight junction molecules *Tjp1, CLDN1, OCLN*, and *CLDN3* in small intestine tissues of mice were evaluated by qRT-PCR. **(B)** Experimental model of fractionated and single radiation exposure in Caco-2 cells, mimicking *in vivo* radiation exposure conditions. **(C)** ROS production in Caco-2 cells after fractionated and single radiation exposure. ROS levels were analyzed by H_2_DCFDA (20 μM) staining and imaging flow cytometry. **(D)** mRNA expression levels of tight junction molecules *Tjp1, OCLN*, and *CLDN1* in Caco-2 cells from the Con, Fraction, and Single groups, evaluated by qRT-PCR. **(E)** Immunofluorescence staining of the tight junction protein ZO-1 in Caco-2 cells following fractionated and single radiation exposure in the Con, Fraction, and Single groups, magnification= 400x. Data are presented as the mean ± standard deviation. Significant differences are indicated as ***p < 0.001, **p < 0.01, *p < 0.05 between groups. Con, non-irradiated control group; Fraction, fractionated radiation exposure group; Single, single radiation exposure group. ns = not significant.

To investigate the effects of fractionated and single radiation exposures on the intestinal tight junction barrier, we used *in vivo* and *in vitro* models. In the small intestinal tissue of irradiated mice, the mRNA levels of the tight junction molecules *Tjp1, CLDN1, CLDN3*, and *OCLN* were considerably reduced in the Single group compared to those in the Con group (**Figure 6A**). Additionally, mRNA levels of tight junction molecules in the Single group were markedly lower than those in the Fraction group. The mRNA expression levels of *CLDN1* were not significantly different between the Con and fraction groups. To replicate the *in vivo* radiation effects, Caco-2 cells were exposed to either four fractionated irradiations or a single dose of irradiation within one passage. Radiation-induced cellular responses were confirmed (**Figure 6B**). ROS production was assessed using H_2_DCFDA staining and flow cytometry, revealing markedly higher fluorescence intensity in the Single group compared to that in the Fraction group, indicating increased oxidative stress (**Figure 6C**). Consistent with the *in vivo* findings, the Single group exhibited markedly reduced mRNA expression of tight junction-related genes (*Tjp1, OCLN*, and *CLDN1*), compared to the Fraction group. Additionally, *Tjp1* and *OCLN* expression was markedly reduced in both radiation-exposed groups relative to the Con group, whereas *CLDN1* expression did not exhibit a significant difference (**Figure 6D**).

Immunofluorescence analysis of ZO-1 further revealed a marked decrease in ZO-1 expression following single radiation exposure compared to fractionated exposure (**Figure 6E**). These findings suggest that single radiation exposure induces oxidative stress and disrupts the tight junction barrier, potentially compromising intestinal epithelial integrity.

### Exogenous SCFA supplementation mitigates radiation-induced intestinal barrier dysfunction

**Figure 7:**
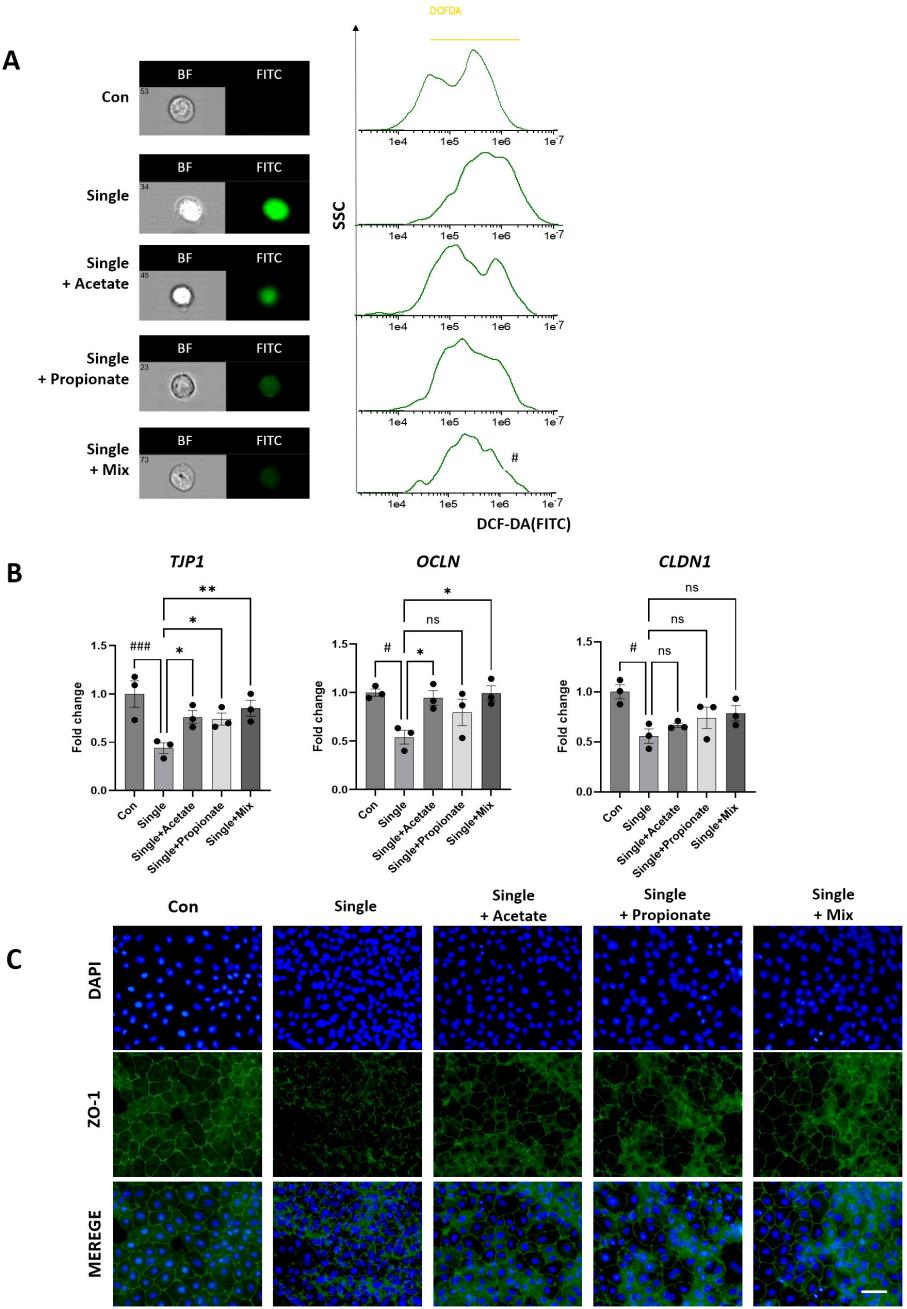
Analysis of tight junction integrity after irradiation and SCFA treatment in differentiated Caco-2 monolayers. **(A)** ROS levels were measured by H_2_DCFDA staining and imaging flow cytometry following irradiation and SCFA treatment (acetate, propionate, or Mix [2 mM each, 24 h]). **(B)** mRNA expression of tight junction molecules (*Tjp1, OCLN, CLDN1*) was evaluated by qRT-PCR in Con, Single, Single+Acetate, Single +Propionate, and Single+Mix groups. **(C)** Representative immunofluorescence images showing ZO-1 staining (green) and nuclei (DAPI, blue), magnification=400x. illustrating changes in tight junction localization and integrity after irradiation and SCFA treatment. Data are presented as mean ± standard deviation. Statistical significance is indicated as ###p < 0.001, #p < 0.05 *vs*. Con; **p < 0.01, *p < 0.05 *vs*. Single. ns, not significant. Con, non-irradiated control group; IR, Single, single radiation exposure group.

Given the marked reduction of acetate and propionate levels observed in the Single group (**Figure 4**), Caco-2 cells were treated with exogenous acetate or propionate following single-dose irradiation. ROS measurement using H_2_DCF-DA staining showed pronounced ROS accumulation in the Single group, which was markedly reduced by acetate and propionate supplementation (**Figure 7A**). RT-PCR analysis demonstrated that the Single group exhibited decreased expression of *Tjp1, OCLN*, and *CLDN1* to 0.45–0.65 fold of control. Supplementation with acetate or propionate increased these levels by 1.3–1.6 fold relative to the Single group, with the Single+Mix condition restoring *Tjp1* to 2.0-fold, comparable to the Single group (**Figure 7B**). Furthermore, immunofluorescence analysis revealed that ZO-1 expression, disrupted in the Single group, was partially restored along the cell membrane in acetate and propionate-treated groups (**Figure 7C**). Collectively, these results demonstrate that exogenous SCFA supplementation attenuates radiation-induced tight junction disruption and ROS accumulation. In addition, SCFA treatment partially restored ZO-1–based epithelial barrier integrity.

## Discussion

Radiation exposure is well known to affect the gut microbiota by altering the intestinal microenvironment and inducing the release of inflammatory cytokines. However, research on the differential effects of various exposure types, such as single and fractionated radiation exposure, remains insufficient (Gerassy-Vainberg et al., 2018). Studies on radiation workers who have been chronically exposed to radiation over long periods, either because of radiation accidents or low-dose radiation, highlight the importance of microbiome balance and long-term health implications. Further research is needed to evaluate the cumulative biological effects of different types of radiation exposure (Tang, 2024). In this study, we established an animal model to investigate the functional consequences of radiation-induced gut dysbiosis. By comparing the effects of single and fractionated radiation exposures, we assessed alterations in microbial diversity and SCFA metabolism, providing insights into how different exposure regimens influence gut microbial function and host-microbiome interactions.

Our findings indicate that single radiation exposure elicits stronger inflammatory responses than fractionated exposure (**Figure 1**). This was evident from the significant increase in DNA damage markers, as indicated by γ-H2AX foci numbers. γ-H2AX foci formation is widely recognized as a sensitive and quantitative biomarker for DNA double-strand breaks following radiation exposure (Mariotti et al., 2013). Previous studies have demonstrated a correlation between γ-H2AX foci formation and radiation dose, underscoring its role as a key indicator of radiation-induced genotoxic stress (Lee et al., 2022). CBC analysis further demonstrated that single radiation exposure had a greater impact on CBC parameters than fractionated exposure, suggesting a more pronounced hematopoietic and systemic inflammatory response. Previous studies have linked radiation-induced CBC alterations to immune suppression and oxidative stress, which may exacerbate tissue damage (Zhou et al., 2024). Histological analysis of the small intestine revealed remarkable structural damage in the single radiation group, characterized by shortened villi and increased crypt depth. These findings align with previous studies indicating that acute radiation exposure induces oxidative stress, exacerbating inflammation and tissue injury (Lu et al., 2023). Among the inflammatory cytokines analyzed (IL-6, MCP-1, IL-1β), only IL-6 exhibited a statistically significant difference between the Single and Fraction groups. IL-6 is a highly radiation-sensitive cytokine and plays a key role in the early inflammatory response following radiation exposure (Schaue et al., 2012). These findings suggest that, although radiation exposure generally induces inflammatory and tissue damage responses, the severity of these effects varies depending on the exposure type. Our study demonstrated that single radiation exposure causes more severe damage than fractionated exposure, emphasizing the importance of understanding dose fractionation effects in radiation biology.

Several clinical studies have reported that patients undergoing abdominal or pelvic radiotherapy exhibit notable alterations in gut microbiota, including reduced alpha diversity and shifts in the *Firmicutes/Bacteroidota* ratio (Wang et al., 2021; Nam et al., 2013; Montassier et al., 2021). These findings are consistent with our observations in irradiated mice, particularly in the single-dose group, where significant dysbiosis and reduced microbial richness were evident (**Figure 2**). Single radiation exposure induced remarkable changes in microbial diversity and composition, characterized by an increase in *Firmicutes* (F) and a decrease in *Bacteroidota* (B), leading to an increase in the F/B ratio, which is an indicator of gut microbiota health. An increased F/B ratio has been associated with metabolic disturbances, inflammation, and gut dysbiosis in various studies, suggesting that radiation exposure may disrupt microbial homeostasis at the phylum level and contribute to adverse physiological effects (Magne et al., 2020). At the genus level, the observed increase in *Lachnospiraceae* and decrease in *Muribaculaceae* suggest that single irradiation may create a microbial environment that favors inflammatory responses. *Lachnospiraceae*, known for its role in SCFA production, is often associated with energy harvesting and metabolic disorders when its abundance is dysregulated (Vacca et al., 2020). Conversely, *Muribaculaceae* spp., predominant members of the *Bacteroidota* phylum, play crucial roles in maintaining gut barrier integrity and modulating immune responses. Its decline has been linked to increased susceptibility to intestinal inflammation (Zhu et al., 2024). Analysis of alpha diversity revealed that the single radiation exposure group exhibited the most notable decrease in the Chao1, Shannon, and Simpson diversity indices. This indicates that single radiation exposure markedly reduced gut microbial diversity, exerting a greater impact on the microbial balance compared to fractionated exposure. Furthermore, beta diversity analysis, visualized using principal coordinate analysis, demonstrated distinct microbial community variations depending on the type of radiation exposure (Lavrinienko et al., 2018). Notably, the Single group showed a more dispersed microbial community structure than the Con and Fraction groups, with some samples displaying substantial differences compared to the Con group. These findings highlight that single radiation exposure induces pronounced alterations in microbial composition, thereby increasing the likelihood of gut microbial dysbiosis. However, one limitation of this study is the absence of a 9-week-old non-irradiated control group, which makes it difficult to clearly distinguish age-related effects from radiation-induced changes. Although a 24-week-old control group was included as a reference for both irradiated groups, baseline physiological and microbial differences between 9- and 24-week-old mice may still have contributed to the observed outcomes.

Furthermore, LEfSe-based LDA analyses were performed to compare each Single group with the non-irradiated Control. The Single group displayed significantly altered microbiota relative to Controls, including increased abundance of inflammation-associated taxa such as *Desulfovibrio, Desulfovibrionaceae*, and *Desulfovibrionia*. In contrast, the Fractionated group did not show statistically significant differential taxa compared with Controls, supporting the conclusion that single-dose exposure induces more pronounced gut dysbiosis (**Supplementary Figure 3A**). Extending this analysis, direct comparison between single and fractionated irradiation groups revealed significant differences in microbial composition (*p* < 0.05, **Figure 3**). The single exposure group exhibited a higher relative abundance of *Firmicutes* and *Lactococcus*, with *Desulfovibrionaceae* and *Desulfobacterota*, SRB known to contribute to gut inflammation and epithelial barrier dysfunction, also being more prevalent (Rajilić-Stojanović and De Vos, 2014; Zhang et al., 2019). In contrast, the fractionated exposure group exhibited a higher abundance of *Bacteroidota, Bacteroidaceae, Prevotellaceae, Rikenellaceae*, and *Alistipes*, many of which produce SCFAs that help regulate gut homeostasis and immune function (Koh et al., 2016; Ríos-Covián et al., 2016). These results suggest that single radiation exposure induces a microbiome shift favoring pro-inflammatory taxa, whereas fractionated radiation exposure supports SCFA-producing bacteria, potentially mitigating radiation-induced gut dysbiosis.

SCFAs—particularly acetate, propionate, and butyrate are key microbial metabolites that influence host metabolism, immune regulation, and intestinal barrier function (Liu et al., 2023). SCFAs strengthen gut epithelial integrity by enhancing tight junction protein expression and suppressing pro-inflammatory responses (Silva et al., 2020). Our SCFA analysis revealed a marked reduction in acetic and propionic acid levels in the Single exposure group compared to those in the Con group, whereas no significant differences were observed between the Con and Fraction groups (**Figure 4**). Butyrate levels remained unchanged across all groups, consistent with previous studies suggesting that butyrate levels are less sensitive to radiation-induced gut microbiota shifts (Louis and Flint, 2017). One of the key biological functions of SCFAs is to activate GPCRs, such as GPR41, GPR43, and GPR109a, which play essential roles in maintaining host immune responses and gut barrier integrity (Morrison and Preston, 2016). Reduced activation of GPR41 and GPR43 is associated with increased intestinal permeability and systemic inflammation (Macia et al., 2015). Moreover, recent studies suggest that SCFAs, particularly acetate and propionate, play a crucial role in regulating intestinal immune responses via GPR41 and GPR43. These microbial metabolites modulate pro-inflammatory cytokine expression, including IL-6 and IL-1β, through GPR-mediated signaling pathways (Guo et al., 2020; Ge et al., 2024; Gerassy-Vainberg et al., 2018). In our study, the depletion of SCFAs and reduced GPR41/43 expression observed in irradiated mice may have contributed to the elevated cytokine levels, indicating a possible mechanistic link between microbial dysbiosis and immune dysregulation. Consistently, the Single exposure group exhibited markedly lower expression levels of GPR41 and GPR43 compared to the Con and Fraction groups, whereas GPR109a expression remained unaffected. These findings indicate that single radiation exposure not only reduces SCFA production but also impairs SCFA-sensing mechanisms, potentially exacerbating gut dysfunction and systemic inflammatory responses. Specifically, we performed correlation analyses to identify which differentially abundant bacterial taxa (*Muribaculaceae, Alistipes*) were associated with SCFA production, including key metabolites (acetate, propionate) and SCFA-sensing receptors (GPR41, GPR43, GPR109). As shown in **Figure 5**, these analyses revealed that fractionated irradiation partially preserves the microbiota–metabolite–immune signaling axis, whereas single-dose irradiation markedly disrupts these correlations. This disruption may explain the stronger inflammation seen after single-dose exposure and shows how different radiation regimens can differently influence host responses.

To further elucidate the effects of radiation exposure on gut epithelial integrity, Caco-2 cells were subjected to either fractionated or single-dose irradiation (**Figure 6**). ROS production, a key factor in oxidative stress, was assessed using H_2_DCFDA staining and imaging flow cytometry. The Single radiation exposure group showed considerably higher fluorescence intensity compared to the Fraction group, indicating increased oxidative stress. Oxidative stress is a major contributor to radiation-induced tissue damage and disrupts gut barrier function by reducing the expression of tight junction proteins. Consistent with this finding, mRNA expression analysis demonstrated that tight junction-related genes (*Tjp1, OCLN*, and *CLDN1*) were markedly downregulated in the Single group. Additionally, immunofluorescence staining confirmed a marked decrease in ZO-1 expression following single radiation exposure, indicating tight junction disruption and compromised epithelial barrier function. The observed decrease in ZO-1 and tight junction-related gene expression in the Single group further supports the role of the gut microbiota and its metabolites in maintaining intestinal integrity and protecting against radiation-induced damage (Shukla et al., 2016).

Furthermore, our functional experiments confirmed that exogenous supplementation with acetate or propionate can partially rescue tight junction integrity (*Tjp1, OCLN*) and oxidative stress in irradiated intestinal epithelial cells. These findings suggest that radiation-induced loss of SCFA-producing bacteria and subsequent reduction in SCFAs directly contribute to barrier dysfunction, rather than being a mere correlative change. Importantly, the reduction of SCFAs and associated epithelial barrier dysfunction observed in our mouse model also reflects clinical features of radiation enteritis, highlighting the translational relevance of our findings (Li et al., 2021; Yang et al., 2023). Consistent with this, previous studies have reported that SCFAs, particularly acetate and propionate, promote the expression of tight junction proteins in intestinal epithelial cells and alleviate barrier dysfunction (Diao et al., 2019; Andrani et al., 2023). This provides a mechanistic link connecting microbiota alterations, metabolic shifts, and intestinal barrier impairment, highlighting the pivotal role of the microbiota–SCFA–barrier axis in radiation-induced gastrointestinal injury.

## Conclusion

**Figure 8:**
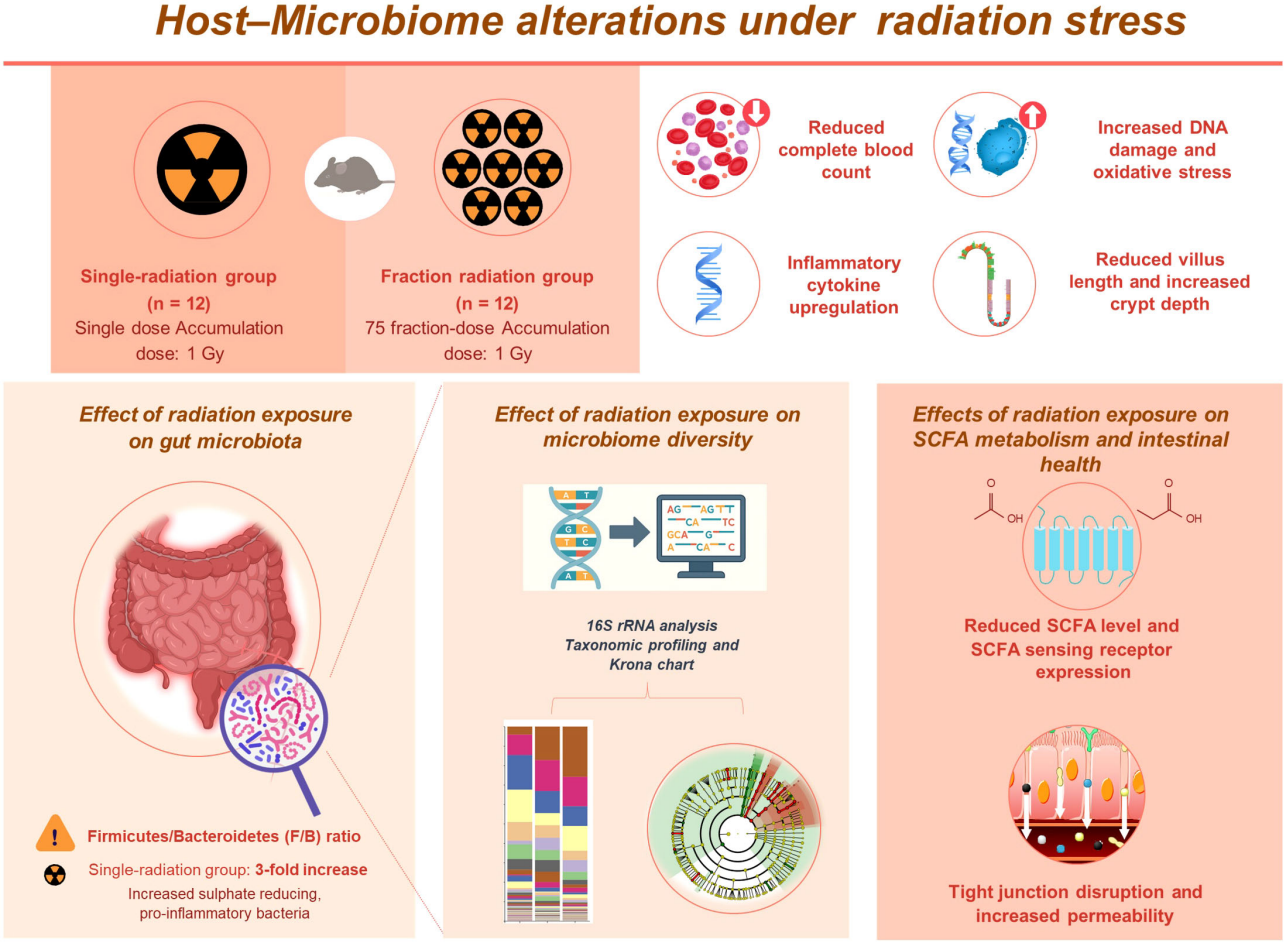
Schematic diagram summarizing the differential effects of single and fractionated radiation exposure on the gut ecosystem and intestinal barrier. Single-dose exposure led to greater systemic and intestinal damage, including increased inflammatory responses and oxidative stress. Gut microbiota analysis showed a higher *Firmicutes/Bacteroidetes* ratio and enrichment of pro-inflammatory bacteria. SCFA levels and receptor expression were reduced, along with tight junction gene expression. These findings suggest that single radiation exposure causes more pronounced gut dysbiosis and barrier disruption than fractionated exposure.

This study provides novel insights into the differential effects of single and fractionated radiation exposures on the gut microbiota composition and intestinal health. Our findings demonstrate that single radiation exposure induces gut microbiota dysbiosis, considerably reduces SCFA production and SCFA-sensing receptor expression, and exacerbates intestinal barrier dysfunction compared to fractionated radiation exposure (**Figure 8**). These findings emphasize the critical role of exposure modality in modulating microbiota-mediated host responses and suggest that microbiota-targeted strategies could offer therapeutic potential in mitigating radiation-induced gastrointestinal dysfunction. However, our model with a cumulative 1 Gy dose does not fully capture chronic low-dose exposures relevant to occupational settings, and interspecies differences limit direct translation to humans. Moreover, the therapeutic potential of SCFA supplementation under prolonged low-dose radiation remains speculative. Nevertheless, caution should be taken when extrapolating from murine models to humans due to fundamental differences in baseline microbiota composition, immune system complexity, and radiation sensitivity. Future studies with longitudinal and human validation will be necessary to address these limitations and to explore microbiota-targeted interventions, such as SCFA supplementation or probiotics, as potential therapeutic strategies.

## Data Availability

The raw sequencing data have been deposited in the NCBI Sequence Read Archive (SRA), BioProject ID PRJNA1253932.
