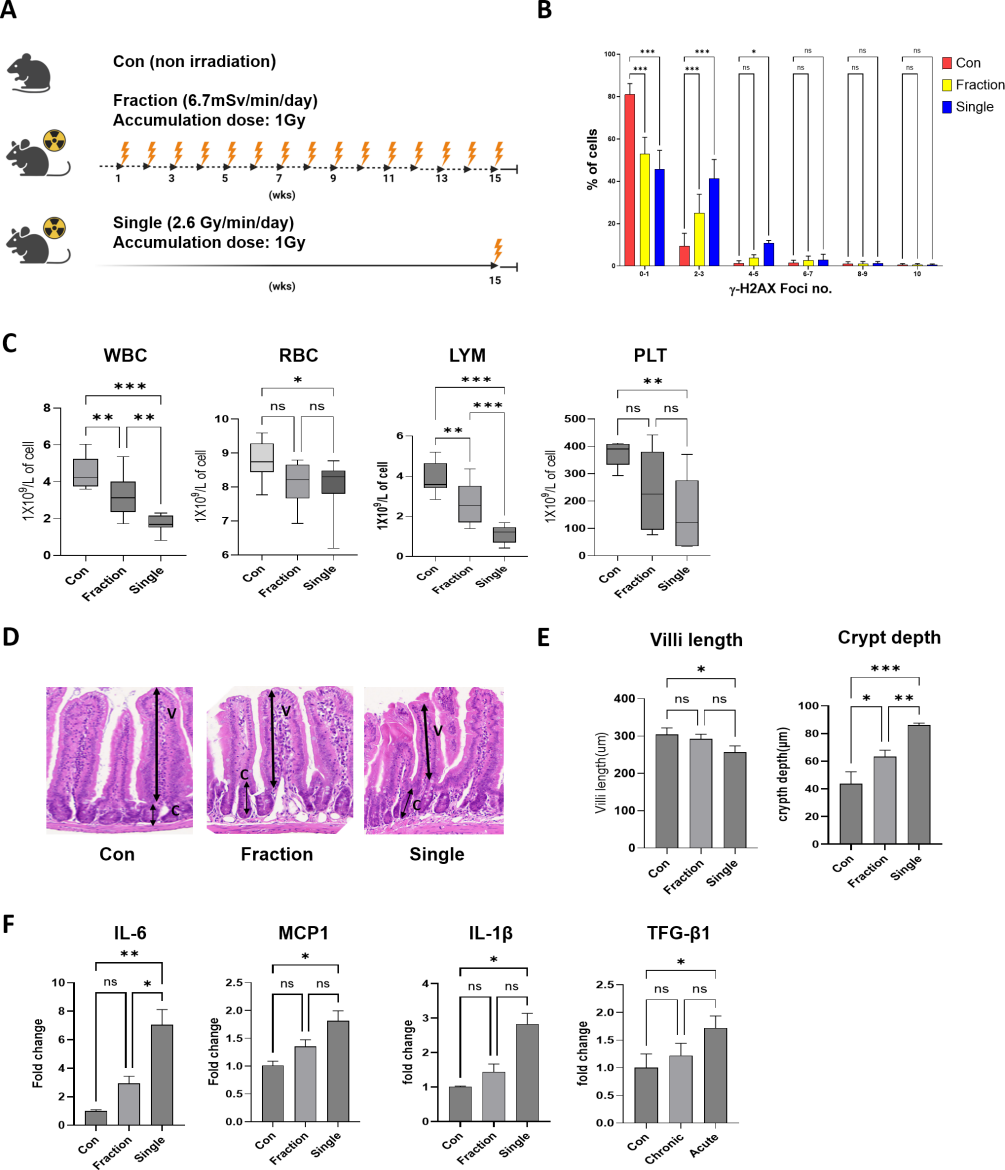# Correction: Single radiation exposure induces gut microbiota dysbiosis and decreases short-chain fatty acid metabolism and intestinal barrier integrity in mice

**DOI:** 10.3389/fcimb.2025.1769533

**Published:** 2026-01-05

**Authors:** Mineon Park, You Yeon Choi, Yanghee Lee, Minsu Cho

**Affiliations:** 1 Laboratory of Biological Dosimetry, National Radiation Emergency Medical Center, Korea Institute of Radiological & Medical Sciences (KIRAMS), Seoul, Republic of Korea; 2 Department of Convergence Korean Medical Science, College of Korean Medicine, Kyung Hee University, Seoul, Republic of Korea

**Keywords:** single and fractionated radiation responses, gut microbiota, SCFAs, intestinal barrier dysfunction, dysbiosis

Author “Yanghee Lee” was erroneously assigned as equal contributing author. “Mineon Park” and “You Yeon Choi” have co–first authorship.

The original version of this article has been updated.

There was a mistake in **Figure 1B** as published. The numbers on the x-axis (horizontal axis) and typo error were incorrectly indicated. The corrected **Figure 1B** appears below.

**Figure 1:**