# Supplementary material for: Single radiation exposure induces gut microbiota dysbiosis and decreases short-chain fatty acid metabolism and intestinal barrier integrity in mice
Source: Front Cell Infect Microbiol. 2025 Sep 17;15:1654976. doi: 10.3389/fcimb.2025.1654976 (PMC12484129; doi:10.3389/fcimb.2025.1654976)
Supplement: Supplementary file 1 [file DataSheet1.docx]

**Supplementary Materials**


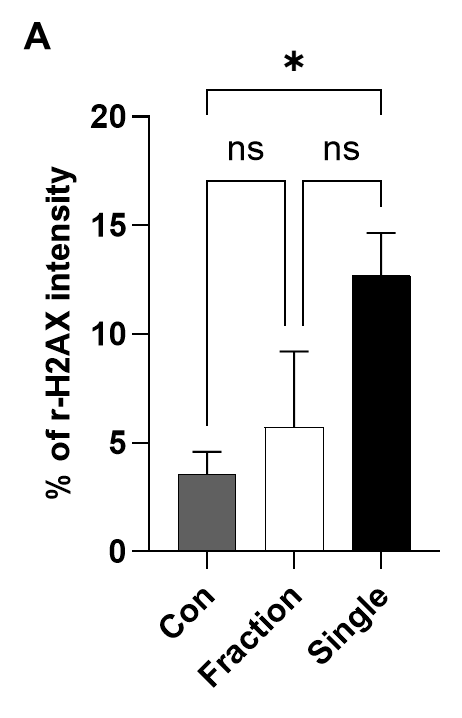


**Figure S1. Representative distribution of γ-H2AX fluorescence intensity in mouse peripheral blood mononuclear cells** (A) Analysis of γ-H2AX intensity in mouse peripheral blood monocytes using imaging flow cytometry after fractionated and single radiation exposure. Data are presented as the mean ± standard deviation (n = 3/group). Significant differences are indicated as **p* < 0.05 between groups. Con, non-irradiated control group; Fraction, fractionated radiation exposure group; Single, single radiation exposure group.


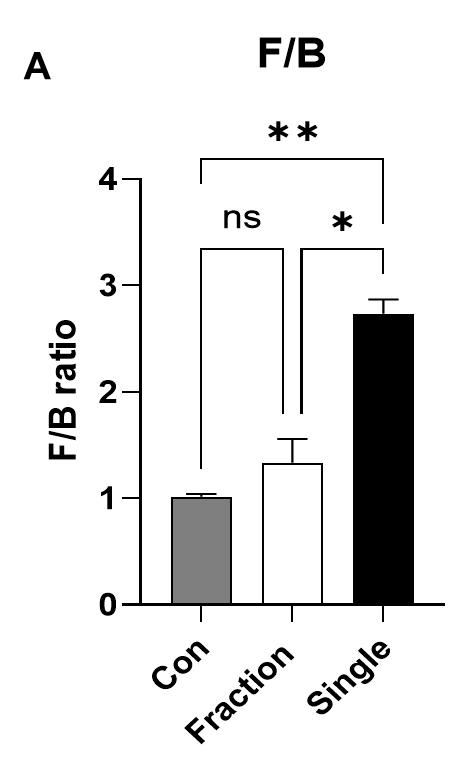


**Figure S2. Phylum-level microbial composition ratio of Firmicutes and Bacteroides in the mice fecal samples.** (A) The relative abundances of *Firmicutes* and *Bacteroidota* at the phylum level were analyzed in fecal samples from mice in the Con, Fraction, and Single groups based on 16S rRNA gene sequencing data. Significant differences are indicated as ***p* < 0.01, **p* < 0.05 between groups. Con, non-irradiated control group; Fraction, fractionated radiation exposure group; Single, single radiation exposure group.

**
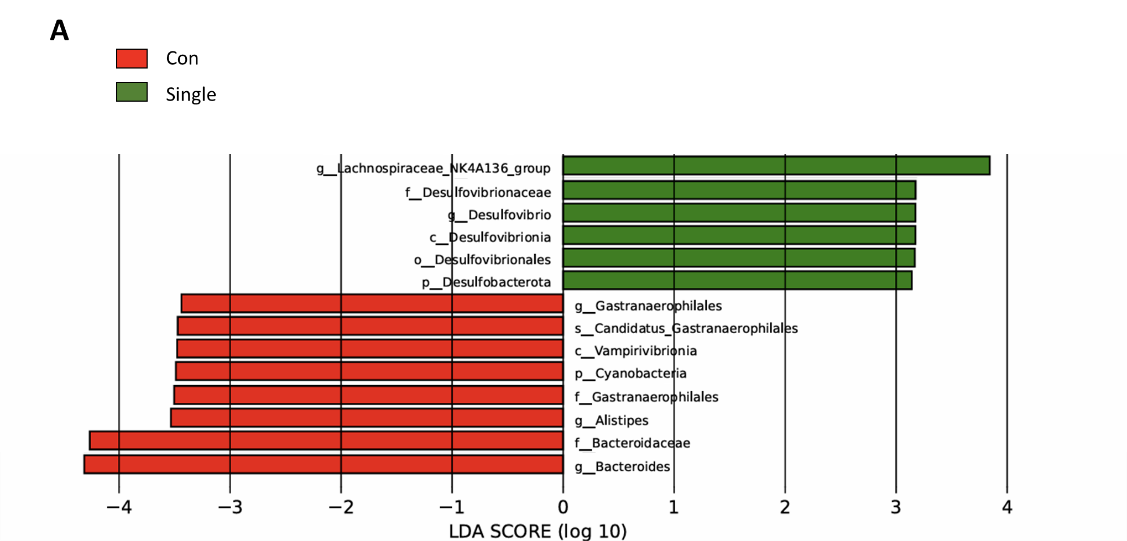
**

**Figure S3.** **Comparison of differentially abundant microbial taxa in the fecal samples from Con and Single radiation-exposed mice.** The LEfSe-based LDA score plot shows differentially abundant microbial taxa in fecal DNA between the Con and Single radiation exposure groups. Red bars represent taxa enriched in the Con group, while green bars indicate taxa enriched in the Single group.
